# Diagnostic Pitfalls of Digital Microscopy Versus Light Microscopy in Gastrointestinal Pathology: A Systematic Review

**DOI:** 10.7759/cureus.17116

**Published:** 2021-08-12

**Authors:** Wangpan Shi, Petros Georgiou, Aqsa Akram, Matthew C Proute, Tatsiana Serhiyenia, Mina E Kerolos, Roshini Pradeep, Nageshwar R Kothur, Safeera Khan

**Affiliations:** 1 Pathology, California Institute of Behavioral Neurosciences & Psychology, Fairfield, USA; 2 Internal Medicine, California Institute of Behavioral Neurosciences & Psychology, Fairfield, USA; 3 Department of Oncology, Oxford University, Oxford, GBR; 4 General Medicine, California Institute of Behavioral Neurosciences & Psychology, Fairfield, USA

**Keywords:** digital microscopy, diagnosis, diagnostic pitfalls, gastrointestinal pathology, whole slide imaging, digital slide

## Abstract

Digital microscopy (DM) is one of the cutting-edge advances in pathology, which entails improved efficiency, diagnostic advantages, and potential application in virtual diagnosis, particularly in the current era of the coronavirus disease (COVID-19) pandemic. However, the diagnostic challenges are the remaining concerns for its wider adoption by pathologists, and these concerns should be addressed in a specific subspecialty. We aim to identify the common diagnostic pitfalls of whole slide imaging (WSI), one modality of DM, in gastrointestinal (GI) pathology. From validating studies of primary diagnosis performance, we included 16 records with features on GI cases involved, at least two weeks wash-out periods, and more than 60 case study designs. A tailored quality appraisal assessment was utilized to evaluate the risks of bias for these diagnostic accuracy studies. Furthermore, due to the highly heterogeneous studies and unstandardized definition of discordance, we extract the discordant cases in GI pathology and calculate the discrepant rate, resulting from 0.5% to 64.28%. Targeting discrepancy cases between digital microscopy and light microscopy, we demonstrate five main diagnostic pitfalls regarding WSI as follows: additional time to review slides in WSI, hard to identify dysplasia nucleus, missed organisms like *Helicobacter pylori* (*H. pylori*), specific cell recognitions, and technical issues. After detailed reviews and analysis, we generate two essential suggestions for further GI cases signing out by DM. One is to use systematized 20x scans for diagnostic workouts and requesting 40x or even 60x scans for challenging cases; another is that a high-volume slides training should be set before the real clinical application of WSI for primary diagnosis, particularly in GI pathology.

## Introduction and background

Since the outbreak of coronavirus disease (COVID-19) across the world, the manner in which doctors practice medicine has significantly changed, especially the need for telemedicine through digital devices [1]. It has been observed that during the COVID-19 pandemic, about two-thirds of in-person doctor visits have been replaced by telehealth in the USA [2]. Additionally, other digital health technologies like wireless medical devices and software as medical devices are promoted and regulated by the Food and Drug Administration (FDA) for clinical applications [3]. The whole idea of digitalization is to promote efficiency, which attracts pioneers in the realm of pathology. Digital pathology (DP) is a newly developed technology that involves the scanning of traditional slides to create digital images and whole slide imaging (WSI) modality, which is the most widely adopted way for pathologists to diagnose, educate, and research [4]. The major advantages of utilizing digital microscopy (DM) are well identified as reduced risk of patients' slide damage, flexible sign-out mode, and better collaboration of pathologists in a challenging case [5].

As for primary diagnosis, the WSI system needs to be meticulously verified through validation studies conducted in their institutions. To date, only one type of digital pathology device has been approved by the FDA for primary diagnostic purposes [6]. A consensus guideline was developed by the College of American Pathologists Pathology and Laboratory Quality Center (CAP-PLQC) in 2013 to guide laboratories for this validation process, which highlights the following study methodology: at least 60 cases of sample inclusion, minimum two weeks wash-out period, and a real clinical application setting. Also, the intraobserver concordance rate, which is the agreement rate between two diagnoses by digital versus glass microscopy, should be established by the same previously trained pathologist [7]. A recently updated guideline, published in 2020, differs from the 2013 guideline in terms of collaborative organizations and revision processes. Most important of all, although not evidence-based, the new guideline suggests that pathologists should read slides in random order during the entire validation process [8].

Regardless of the guideline, several validation studies have been conducted over the past 10 years, which favorably report the diagnostic concordance rate from 87% to 98.3% [9]. However, the satisfying results of agreement between digital microscopy and glass microscopy do not eliminate all concerns. In the latest systematic review and meta-analysis by Azam et al., a total of 546 major disagreement cases were identified from 10,410 pathology samples across 25 validation/comparative studies [10]. About half of these discordances are related to evaluating dysplasia, nuclear atypia, or malignancy grading. The next most common reasons for this disagreement are challenging diagnostic cases and finding out small objects [10]. Besides the discordance cases, an inherited factor that hampers the diagnostic ability of WSI is the inability to evaluate structures that need polarization (e.g., amyloid and monosodium urate crystals) [11].

Despite the previous work, no reviewer has addressed the diagnostic pitfalls in a specific subspeciality, and some of the review studies were not conducted based on the CAP-PLQC guidelines. The present review aims to assess validation studies of WSI for primary gastrointestinal (GI) pathology diagnosis to identify the challenges and pitfalls in discordant cases between digital and glass slides.

## Review

Materials and methods

Review Protocol and Question Identification

This present review is designed and conducted following the guidelines by the Preferred Reporting Items for Systematic Review and Meta-Analyses (PRISMA) [12]. The review question identified is "what are the common encountered diagnostic difficulties in gastrointestinal pathology that pathologists are most concerned about when utilizing the WSI mode of digital slides compared with glass slides?" We believe the best answer to this question is to study discordance cases among high-quality validation studies.

Literature Review

To avoid duplicated work and study, the leading researcher did a comprehensive literature review to see if any ongoing, registered, or completed study under the same topic is available. To date, a systematic review called "The diagnostic concordance of whole slide imaging and light microscopy: a systematic review" was registered and published under the protocol CRD42015017859 [13]. It is designed to evaluate an overall concordance rate in digital pathology. Another systematic review was published with the title "The performance of digital microscopy for primary diagnosis in human pathology: a systematic review" under the protocol CRD42018085593, which primarily shows and analyzes the disagreements between digital slides and glass slides [9]. This high-quality review work was published to determine the universal diagnostic concordant and discordant cases in digital pathology [9]. Also, Williams et al. did a comprehensive work on identifying the discrepancy causes in digital microscopy [14]. In cytopathology, Girolami et al. carried out a study on the diagnostic performance and limitations, which features the scanning time and technical challenges as two main obstacles in WSI [15]. Although all of these previously published studies were conducted according to the CAP-PLQC guideline, none of these address the discordant reasons when applying digital microscopy in a specific subspecialty. Due to the well-known variations in different pathology subspecialties in terms of diagnostic protocol, our review is the first study to navigate the diagnostic challenges and the diagnostic discordances of digital microscopy in GI pathology. Besides, we only consider including reliable studies rested on the CAP-PLQC guideline and the updated version in 2021.

Search Strategy

A search of the literature was conducted across the databases: PubMed platform (National Center for Biotechnology Information, US National Library of Medicine, Maryland, USA), Scopus (Elsevier, Amsterdam, The Netherlands), and Embase (Elsevier, Amsterdam, The Netherlands). The search strategy used by the primary researcher is as follows: digital pathology OR whole slide imag* OR virtual microscopy OR digital microscopy OR digital slides OR virtual slides OR telepathology OR telemicroscopy OR digital imag* AND light microscop* OR conventional microscop* OR traditional microscop* OR glass slides OR optical microscop* AND (validation OR validate stud*). Also, preliminary filters were used like full text and validation study, in the last 10 years, humans, and English to narrow down the study pool. Because of the strict need to design a validation study on digital pathology, we deem it unnecessary to search for grey articles to eliminate bias manually.

Demonstrate the Eligibility Criteria

The CAP-PLQC created a highly reliable consensus guideline in 2013, and its updated version was also available in 2021 [7,8]. The Grade A evidence as recommendations is the backbone in our study inclusion and exclusion criteria (Table 1). First, all the validation studies should include trained pathologists, and a complete set of WSI systems is required for primary diagnosis. Second, each study sample set should at least be 60 cases, and a wash-out period of more than two weeks is highly recommended [7,8]. Finally, intraobserver concordance should be established, which pathologists should compare the same pathology specimen reading in digital and glass microscopy rather than taking consensus diagnosis as the standards [16]. Additionally, in the updated version of the guideline, it is suggested to review the digital slides and glass slides randomly. However, we do not believe this is mandatory due to the absence of direct evidence [7,8].

**Table 1:** Inclusion and exclusion criteria These criteria were based on the 2013 guideline posted by the College of American Pathologists Pathology and Laboratory Quality Center and its updated version [7,8].

Inclusion criteria	Exclusion criteria
1. Validation/comparative studies between digital microscopy and light microscopy	1. Utilise digital slides for education or research purposes
2. Primary clinical diagnostic purposes	2. Telepathology
3. Trained pathologists to use whole slide image (WSI) mode	3. No intraobserver concordance was established
4. Each sample set should at least be 60 cases	4. Studies without gastrointestinal pathology cases
5. At least two weeks wash-out period	5. Published in foreign languages
6. Complete components of WSI system for primary diagnosis	6. Non-human pathology specimens involved

Article Screening and Assess for Eligibility

**Figure 1:**
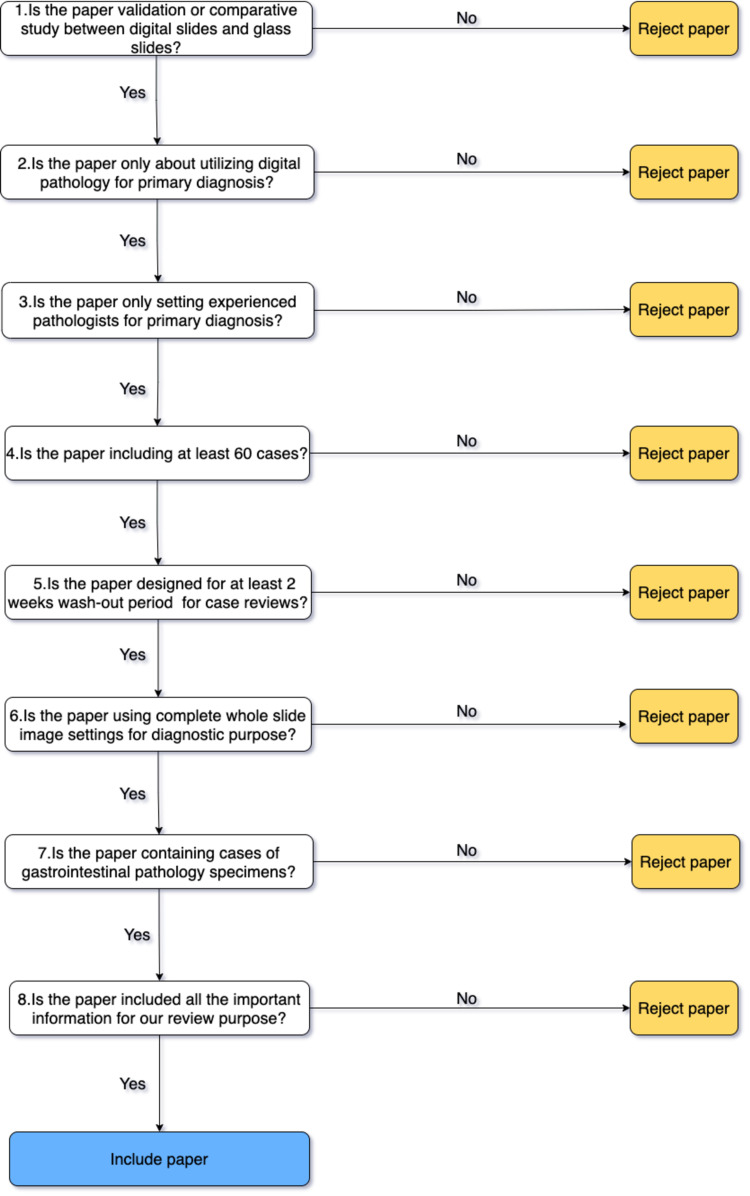
The eligibility assessment algorithm

Independently, two reviewers evaluated the records initially by title or abstract to exclude blatantly unqualified articles. After the process of screening, we further assess the credit of full-text articles by applying the algorithm in Figure 1. The inclusion and exclusion criteria we mentioned above are paralleled with the algorithm to evaluate the eligibility of screened records.

Data Collection and Extraction

Data collection was primarily performed by the leading reviewer based on a specifically tailored data extraction form, in which the key domains are summarized as study design, technical setting, validation methodology, case distribution, and discordant results (Table 2).

**Table 2:** Key domains in data extraction

Domains
Study design
Single/multiple centers, onsite or remote sign-out, retrospective, prospective, or cross-sectional, numbers of pathologists, received training before the study, and included sample number
Technical setting
Scanner use, staining involved, and scanning magnification
Validation methodology
Blinding process, potential bias, wash-out period, with or without a known diagnosis, and clinical information availability
Case distribution
Esophagus, stomach, small intestine, liver, bile duct, pancreas, and large intestine
Discordant result
Discordant rate, reasons for discordance, minor or major discordance, and hazard clinical outcomes

Quality Assessment With Tailored QUADAS-2

The Quality Assessment for Diagnostic Accuracy Studies 2 (QUADAS-2) was utilized to assess the quality of each included study [17]. Our selected studies focused on human GI pathology specimens as samples to compare the digital microscopy (index test) with traditional microscopy (reference standard test) in terms of primary clinical diagnosis. Therefore, we tailored the original QUADAS-2 tool by adding additional signaling questions and excluding signaling questions that do not apply to the present review. Generally, patient selection, index test, reference standard, and flow and timing were allocated as the leading four domains, in which several signaling questions were contained by divided into risk of bias and applicability. We constructed clear instructions for each signaling question and, in each domain, we classified the evaluation category into unclear risk, low risk, and high risk. Not following the updated version of CAP-PLQC guidelines, we believe pathologists who review both the index test and reference standard test in non-random order still have a low risk of bias in domain two. Other than that, more than two negative answers to the signaling questions in each domain will be tagged as high risk of bias. For unclear risk of bias, studies in which essential details are not mentioned or omitted will be classified into this part.

Results

PRISMA Flowchart

**Figure 2:**
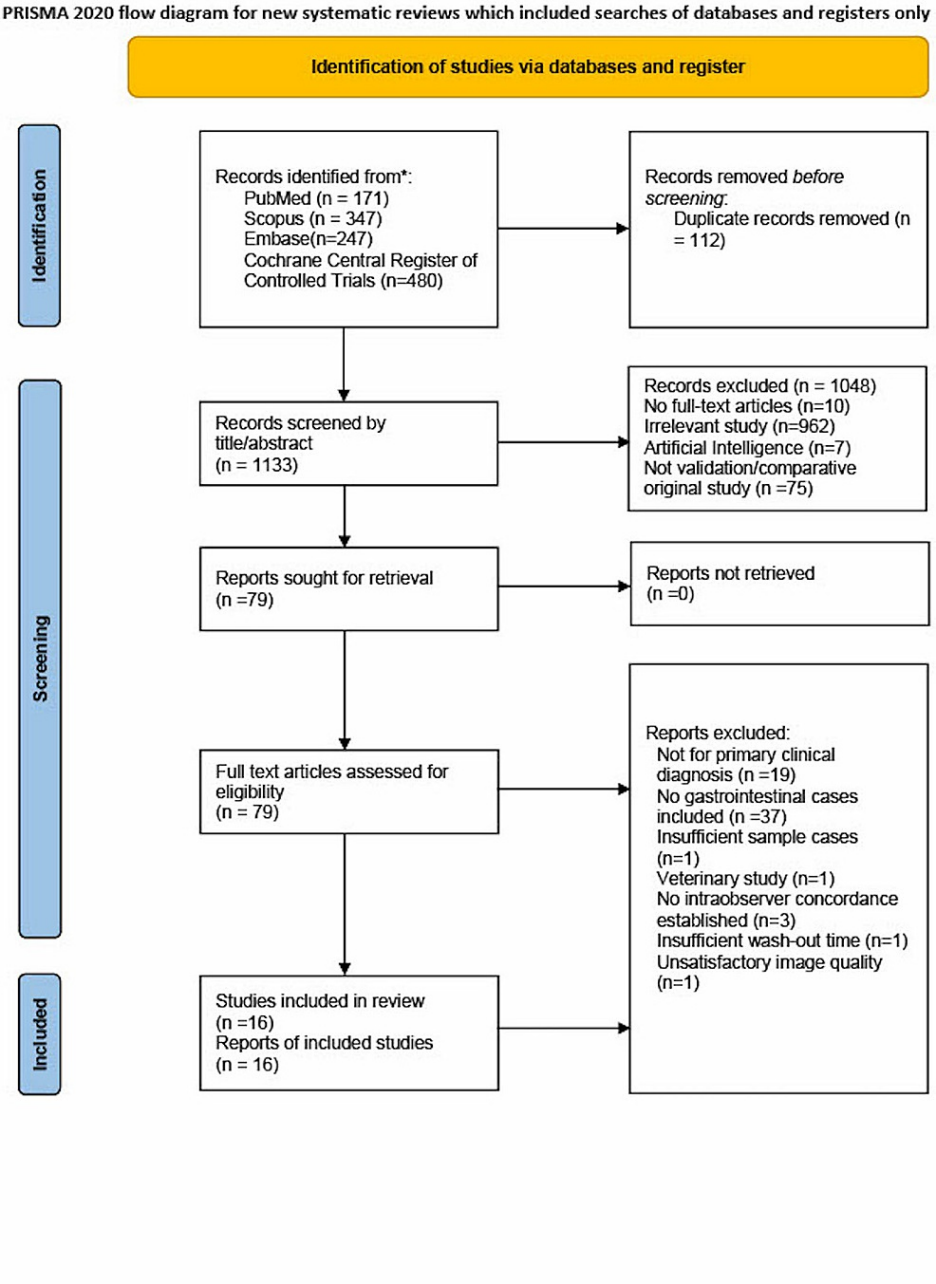
PRISMA 2020 flow diagram PRISMA - Preferred Reporting Items for Systematic Reviews and Meta-Analyses [12].

A total of 1,245 articles were identified by the search strategy we designed. After duplications removal, we reviewed the title or abstract to apply the initial screening process, which only yields 79 studies. Precisely, articles were excluded for any of the following reasons: no full-text articles (n = 10), irrelevant study (n = 962), artificial intelligence (n = 7), and not validation or comparative original study (n = 75). Subsequently, 79 full-text articles were retrieved for further screening, among which fulfill all the requirements were included. They were excluded for the following reasons: not for primary clinical diagnosis (n = 19), no gastrointestinal cases included (n = 37), insufficient sample cases (n = 1), veterinary study (n = 1), no intraobserver concordance established (n = 3), and insufficient wash-out period (n = 1). In the end, we included 16 articles, and all of them are validation studies to evaluate the diagnostic performance of the WSI system. The article selection flow diagram based on Preferred Reporting Items for Systematic Reviews and Meta-Analyses (PRISMA) is demonstrated in Figure 2.

Quality Appraisal of Studies

In summary, due to lack of information, three (18%) studies cannot be assessed for the risk of bias in patient selection, and one study designed by Borowsky et al. features on free cases deferral if the pathologists believe, in real clinical settings, future consultation and additional information are needed [18]. For the risk of bias in the index test part, three (18%) studies included pathology residents and not adequately trained pathologists involved in slides sign out; on top of that, the majority of the studies (65%) do not review digital slides and glass sides randomly. One study did not assess all the selected pathology specimens and was deemed high risk in flow and timing [19]. In our review purpose, we believed the risk of bias in domains of applicability concerns is satisfactory, except studies only focused on the pancreatic, liver, and large intestine pathology specimens [20-22]. Overall, the results are shown in Table 3, and two reviewers conducted the quality appraisal process.

**Table 3:** Tailored QUADAS-2 *Although in the validation study pathologists review the digital slides and glass slides in a fixed sequence, we still regard it as low risk of bias in this category. QUADAS-2, Quality Assessment for Diagnostic Accuracy Studies 2.

Tailored QUADAS-2—the risk of bias and applicability concerns quality appraisal							
Study	RISK OF BIAS				APPLICABILITY CONCERNS		
	PATIENT SELECTION	INDEX TEST	REFERENCE STANDARD	FLOW AND TIMING	PATIENT SELECTION	INDEX TEST	REFERENCE STANDARD
								Al-Janabi et al. 2012 [23]	Unclear	Low*	Low	Low	Low	Low	Low
								Al-Janabi et al. 2013 [24]	Unclear	Low*	Low	Low	Low	Low	Low
Arnold et al. 2015 [25]	Low	Low	Low	Low	Low	Low	Low
Borowsky et al. 2020 [18]	High	Low	Low	Low	Low	Low	Low
Larghi et al. 2019 [20]	Unclear	Low*	Low	Low	Low	Unclear	Low
Loughrey et al. 2015 [26]	Low	High	Low	Low	Low	Low	Low
Mills et al. 2017 [27]	Low*	Low	Low	Low	Low	Low	Low
Mukhopadhyay et al. 2017 [28]	Low	Low	Low	Low	Low	Low	Low
Rao et al. 2021 [29]	Low	Low*	Low	Low	Low	Low	Low
Saco et al. 2017 [21]	Low	Low*	Low	Low	Low	Unclear	Low
Samuelson et al. 2021 [30]	Low	Low*	Low	Low	Low	Low	Low
Snead et al. 2015 [31]	Low	Low*	Low	Low	Low	Low	Low
Tabata et al. 2017 [32]	Low	Low*	Low	Low	Low	Low	Low
Thrall et al. 2015 [33]	Low	High	Low	Low	Low	Low	Low
van der Post et al. 2013 [22]	Low	High	Low	Low	Low	Unclear	Low
Villa et al. 2017 [34]	Low	Low*	Low	Low	Low	Low	Low

Discussion

Characteristics of Digital Microscopy Validation Studies of GI Pathology

Because the WSI system is listed as the highest risk of medical device (class III) under regulation, we deem it necessary to conduct this review for a better clinical application of digital microscopy, especially in the subspecialty of GI pathology [35]. As shown in Tables 4, 5, validation or comparative studies of digital microscopy with glass microscopy were conducted from 2012 to the present. Due to the meticulous criteria of the methodology for primary diagnosis recommended by the College of American Pathologists [7], it is noted that most of the studies were designed in the USA or Europe. At least two experienced pathologists, an average of 14, partake in those studies, and the included GI sample number also varies. The majority of the investigators use a 20x scanning magnification for conventional study slides, and 40x scans, mixed case by case.

**Table 4:** Study characteristics of included studies GI, gastrointestinal.

Author, year	Location	Study design	Pathologists number	GI sample category	GI sample
Al-Janabi et al. 2012 [23]	Netherlands	Validation	20	All	100
Al-Janabi et al. 2013 [24]	Netherlands	Validation	3	All	66
Arnold et al. 2015 [25]	USA	Validation	N/A	All	13
Borowsky et al. 2020 [18]	USA	Comparative	19	All	523
Larghi et al. 2019 [20]	Italy	Comparative	5	Pancreas	60
Loughrey et al. 2015 [26]	UK	Validation	3	All	100
Samuelson et al. 2021 [30]	USA	Validation	5	All	31
Mills et al. 2017 [27]	USA	Comparative	2	All	200
Mukhopadhyay et al. 2017 [28]	USA	Comparative	16	All	523
Saco et al. 2017 [21]	Spain	Validation	24	Liver	176
Snead et al. 2015 [31]	UK	Validation	17	All	405
Tabata et al. 2017 [32]	Japan	Validation	9	All	615
Thrall et al. 2015 [33]	USA	Validation	57	All	25
Rao et al. 2021 [29]	India	Validation	18	All	122
Villa et al. 2017 [34]	France	Validation	3	All	41
van der Post et al. 2013 [22]	Netherlands	Validation	4	Large intestine	295

**Table 5:** Study results of included studies GI, gastrointestinal.

Author, year	Scanner magnification	GI sample discrepant rate	Wash-out period	All sample concordant rate	Major discrepancy
Al-Janabi et al. 2012 [23]	X20	5.00%	6 months	95.00%	0
Al-Janabi et al. 2013 [24]	X20	6.06%	1 year	90.00%	2
Arnold et al. 2015 [25]	X20	64.28%	3 months	98.30%	0
Borowsky et al. 2020 [18]	X20	2.91%	31 days	96.36%	15
Larghi et al. 2019 [20]	X20	8.00%	3 months	92.00%	0
Loughrey et al. 2015 [26]	X20	19.00%	6 months	95.30%	1
Samuelson et al. 2021 [30]	X20	29.03%	2 weeks	83.60%	2
Mills et al. 2017 [27]	X20	N/A	6 weeks	99.40%	N/A
Mukhopadhyay et al. 2017 [28]	X40	1.92%	4 weeks	95.10%	10
Saco et al. 2017 [21]	X40	<10%	1.5 months	96.60%	0
Snead et al. 2015 [31]	X40	0.50%	3 weeks	97.70%	2
Tabata et al. 2017 [32]	X20/X40	4.87%	2 weeks	95.60%	8
Thrall et al. 2015 [33]	X20	10.80%	3 weeks	79.00%	0
Rao et al. 2021 [29]	X20/X40	N/A	2 weeks	98.80%	0
Villa et al. 2017 [34]	X40	4.87%	1 month	87.40%	0
van der Post et al. 2013 [22]	X20	10.40%	1 week	89.60%	1

Discrepant Rate and Case Distribution of Digital Microscopy

**Figure 3:**
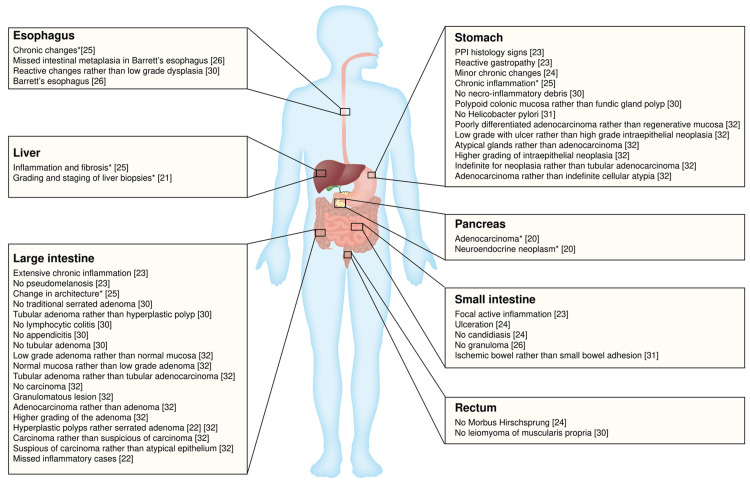
Discrepant cases in GI pathology Specific discrepant cases across the digestive tract are illustrated under the perspective of digital microscopy (DM). Although the consensus diagnosis is not necessarily by light microscopy (LM). *No DM and LM comparative details can be accessed. PPI, proton pump inhibitor; GI, gastrointestinal.

The overall concordance rate of all specialty cases is satisfying from 79.0% to 99.4%; however, the definition of discordance cases was highly heterogenous by each institution, which shows a lowest 0.5% to highest 64.28%. Therefore, we demonstrate those data as GI sample discrepancy rate, which means that different readings were observed between WSI and glass slides. In addition, 15 out of 523 and 10 out of 523 discrepancies entail significant clinical consequences reported by Borowsky et al. and Mukhopadhyay et al., separately [18,28]. The distribution of discrepant cases under the perspective of DM is shown in Figure 3. The stomach and large intestine are the two prime places where disagreements occur, and in parallel with this, the minor difference in grading of the nucleus is also well-reported across those studies as a leading reason.

Diagnostic Pitfalls - Difficulties in Diagnostic Efficiency

By addressing those discordances, we find out that in seven (46.7%) studies, pathologists reported more time consumed in WSI compared with light microscopy (LM). Larghi et al. reported an average of 24 seconds lag between the two slide-view methods [20]. Moreover, a mean of 54 seconds longer of digital pathology was showed by Thrall et al. [33]. This time lag of around one minute could be a significant setback for finishing daily sign-out cases in a large hospital. Despite not being formally recorded, larger resection pathology specimens potentially take more time for reviewing digital slides [27]. For institutions with a large number of pathology samples, this finding could decrease the efficiency and lead to further drawbacks like physician burn-out or financial shortage, which finally affect the diagnostic accuracy. Therefore, the challenge of additional time consuming may render the further implementation of WSI into the daily GI pathology sign-out process.

However, we should not ignore another well-reported phenomenon called the "learning effect," which means the low efficiency in WSI can be overcome by learning more and become experienced [27]. Mills et al. identified that the reading time between digital slides and glass slides is negligible after gaining the reading slide experience of 500 cases [27]. Additionally, lower scanner magnification decreases the slide reading time, but some minor features or bacteria can also be overlooked [31,36]. van der Post et al. point out that image reviewer and laboratory infrastructure software also play critical roles in the diagnostic efficiency of digital pathology [22]. In another part, when it comes to quantitative evaluation of cell numbers due to its clinical relevance, the WSI system excels traditional glass slides in diagnostic efficiency by saving time for manual counting [27].

Even though several studies show a significant challenge of additional time for slide reading, this issue can be well-addressed by making mandatory cases training for pathologists, tailoring the scanner magnification to each case, and standardizing the laboratory infrastructures and protocols regarding the WSI system. Combined with the hidden gain of quantity evaluation, diagnostic efficiency should not be a major concern for adopting digital pathology.

Diagnostic Pitfalls - Difficulties in Evaluation of Nuclear Features

It is suggested by Samuelson et al. that pitfall may occur when grading dysplasia, which relates to evaluating the chromatin details [30]. Specifically, in his study, relative hyperchromasia was unsuccessfully presented in the digital slides for two GI discrepant cases, which fails to diagnose real tubular adenoma [30]. Interestingly, Snead et al. show that two dysplasia cases reported in digital microscopy were missing in light microscopy, which may associate with the darker nuclei seen in DP and observed in Barrett's esophagus by Dr. D Treanor through personal communication [31]. After detailed analysis of the discordant GI cases in Tabata's study, higher grading of adenoma, intraepithelial neoplasia, and carcinoma rather than adenoma are majority cases of disagreement [32]. In fact, due to more mitotic activity of the malignant or high-grade tumor, the nucleus of which is often darker than low grade or benign tumor. Although no direct evidence exists, this over-grading pitfall is potentially correlated with the tendency of darker nuclei in the WSI system mentioned by Snead et al. [31]. It is further proved in a study by Villa et al. that all three pathologists believe they experienced upgrading of dysplasia lesions in DP [34]. One possible explanation is that more weight was given to cellular architecture changes in final diagnosis due to the analytical methodology of reading digital slides compared to LM [27,37]. A solitary case of misdiagnosis of poorly differentiated adenocarcinoma to inflammation was also reported in second diagnostic settings [22]. Among other subspecialty pathology cases, this diagnostic challenge of evaluating nuclear features prevails in 57% of discordance reasons of WSI system in a comprehensive systematic review [38]. Besides, in a routine surgical pathology WSI study, it is suggested that higher magnification of scanner may be a significant factor in helping visualize detailed nuclear features, and further studies are needed to identify the correlation of discordant GI tumor grading with a low magnification of digital microscopy [37]. Therefore, when signing out of GI cases, the pathology department and laboratories should be well aware of this predisposition of darker nucleus change and request higher scanner magnification before utilizing WSI for primary diagnosis.

Diagnostic Pitfalls - Difficulties in Identification of Microorganisms

To date, the most adopted scanner magnification is 20x across those validation studies, which ensues with less scanning time and storage [29]. Nevertheless, according to a study by Al-Janabi et al., *Helicobacter pylori*, *Candida albicans*, and *Giardia duodenalis* are three microorganisms frequently challenging to diagnose under this magnification, and an additional 40x will give them more confidence to identify [23]. Furthermore, another of his study focused on pediatric pathology showed that one missed candidiasis in a small intestine sample under 20x magnification [24]. Low magnification means fewer fine details presented to readers in traditional glass slides, but in most cases, the WSI system can generate a high-quality image in 20x magnification for diagnostic tasks [33]. There are two potential setbacks of resolution of 40x: one is more scanning time and storage, as talked about before, and the other is considered an inherent issue that image clarity is worse at scanning magnification above 20x [29,33]. Thus, in Borowsky et al.'s study design, they request 40x magnification for three slides only to identify *H. pylori* [18]. Subsequently, another study also indicates that the identification of *H. pylori* is still challengeable under 20x magnification, and even the tissue was treated with immunohistochemistry (IHC) [37]. More radically, Snead et al. reported that *H. pylori *could only be identified in the display of 60x, which suggests setting this magnification as default for *H. pylori* gastritis evaluation [31]. Based on the findings reviewed, we believe the best solution to tackle this challenge efficiently is to make GI pathologists informed and educated; when using WSI to read slides looking for microorganisms, a higher than 20x magnification is necessary, but in other cases, this up-gradation is not advisable.

Diagnostic Pitfalls - Difficulties in Specific Cell Identification

There are specific cells with diagnostic power in GI pathology, such as neutrophils in gastric mucosa indicative of acute gastritis and eosinophils in the context of eosinophilic esophagitis. Therefore, identification of those indicative cells is crucial to an accurate diagnosis. It is seen in the study by Arnold et al. that the refractile nature of eosinophilic granules in the cytoplasm gives rise to the challenge to recognize them in digital slides [25]. It is suggested that the color change of scanned slides in WSI compared to glass slides plays a critical role, which could be addressed in future studies by implementing color calibration tools on computer monitors [25]. Additionally, it is reported by Thrall et al. that the small inflammatory cells like neutrophils cannot be identified well under 20x magnification, which is further testified in a study by Bauer and Slaw, which shows better recognition of neutrophils in inflammatory lesions in 40x scans [33,36].

Diagnostic Pitfalls - Difficulties in Technical Settings

Some pathologists expressed the unfamiliarity of the computer mouse to navigate slides is somehow a problem in the diagnostic settings, which is an entirely different experience than traditional microscopes [23]. It takes time for pathologists to learn and become natural in utilizing the WSI system. Moreover, in identifying important chromatic patterns of some instances, basic WSI systems do not hold the ability to capture multiple planes to evaluate the entire thickness of samples, and a function of the Z-stacking feature is often required [18]. Although not related to the present review purpose, similar findings of Z-stacking requirement are also prominent in thick cytologic smears for selective focusing of sample reading [38]. Another critical difficulty encountered by Loughrey et al. is underexposure of image, which makes it impossible counting of intraepithelial lymphocytes in colonic mucosa and hard to differentiate dysplasia [26]. These technical pitfalls can be prevented and tailored case by case through comprehensive quality appraisal of the whole digital diagnostic settings ahead.

Limitation

One limitation of our study is the lack of information in some studies, which intervenes the capability to identify discrepant cases and further analyzes the reasons for diagnostic challenges. Another worth mentioning point is, partially due to the unclear laboratory settings, we do not assess the limitations of the WSI system scanners across studies and they are reported to be a major factor in the diagnostic efficiency and accuracy, especially in studies before 2014 [39].

## Conclusions

Digital pathology is the frontier innovation for the discipline of pathology, of which the benefits and limitations are well-studied in a holistic manner. However, the challenges and potential diagnostic pitfalls that pathologists and researchers seek should be tailored to a particular organ system (e.g., GI pathology). Our study exclusively focused on the process of identifying and analyzing the common difficulties in the discordance cases of WSI with light microscopy among GI pathology. When it comes to the real validating of WSI for primary diagnosis, it is suggested that the significant pitfalls are additional reading time, inclination to hyper-grading of atypia nucleus, missed diagnosis of microorganisms like *H. pylori*, under-identified of granulocytes, and minor technical limitations.

Therefore, in the samples of GI pathology, we recommend pathologists to use the standardized 20x scan for routine diagnostic workouts and request 40x or even 60x scanning for evaluating microorganisms, granulocytes, and challengeable nuclear dysplasia. Besides, implement high volume digital slides sign-out training for pathologists as the general requirement before clinical application of digital pathology and turn to the Z-stacking feature of scanners for better-focused readings in case of thick specimens.

## References

[1] Kichloo A, Albosta M, Dettloff K (2020). Telemedicine, the current COVID-19 pandemic and the future: a narrative review and perspectives moving forward in the USA. Fam Med Community Health.

[2] Patel SY, Mehrotra A, Huskamp HA, Uscher-Pines L, Ganguli I, Barnett ML (2021). Trends in outpatient care delivery and telemedicine during the COVID-19 pandemic in the US. JAMA Intern Med.

[3] (2021). US Food and Drug Administration. What is Digital Health?. https://www.fda.gov/medical-devices/digital-health-center-excellence/what-digital-health.

[4] Farahani N, Parwani A, Pantanowitz L (2015). Whole slide imaging in pathology: advantages, limitations, and emerging perspectives. Pathol Lab Med Int.

[5] Williams BJ, Bottoms D, Treanor D (2017). Future-proofing pathology: the case for clinical adoption of digital pathology. J Clin Pathol.

[6] Food and Drug Administration (2017 (2021). US Food and Drug Administration. FDA allows marketing of first whole slide imaging system for digital pathology. https://www.fda.gov/news-events/press-announcements/fda-allows-marketing-first-whole-slide-imaging-system-digital-pathology.

[7] Pantanowitz L, Sinard JH, Henricks WH (2013). Validating whole slide imaging for diagnostic purposes in pathology: guideline from the College of American Pathologists Pathology and Laboratory Quality Center. Arch Pathol Lab Med.

[8] Evans AJ, Brown RW, Bui MM (2021). Validating whole slide imaging systems for diagnostic purposes in pathology: guideline update from the College of American Pathologists in collaboration with the American Society for Clinical Pathology and the Association for Pathology Informatics. Arch Pathol Lab Med.

[9] Araújo AL, Arboleda LP, Palmier NR (2019). The performance of digital microscopy for primary diagnosis in human pathology: a systematic review. Virchows Arch.

[10] Azam AS, Miligy IM, Kimani PK, Maqbool H, Hewitt K, Rajpoot NM, Snead DR (2021). Diagnostic concordance and discordance in digital pathology: a systematic review and meta-analysis. J Clin Pathol.

[11] Jahn SW, Plass M, Moinfar F (2020). Digital pathology: advantages, limitations and emerging perspectives. J Clin Med.

[12] Moher D, Liberati A, Tetzlaff J, Altman DG (2009). Preferred reporting items for systematic reviews and meta-analyses: the PRISMA statement. PLoS Med.

[13] Goacher E, Randell R, Williams B, Treanor D (2017). The diagnostic concordance of whole slide imaging and light microscopy: a systematic review. Arch Pathol Lab Med.

[14] Williams BJ, DaCosta P, Goacher E, Treanor D (2017). A systematic analysis of discordant diagnoses in digital pathology compared with light microscopy. Arch Pathol Lab Med.

[15] Girolami I, Pantanowitz L, Marletta S (2020). Diagnostic concordance between whole slide imaging and conventional light microscopy in cytopathology: a systematic review. Cancer Cytopathol.

[16] Cornish TC, Swapp RE, Kaplan KJ (2012). Whole-slide imaging: routine pathologic diagnosis. Adv Anat Pathol.

[17] (2021). University of Bristol. QUADAS-2: background document. https://www.bristol.ac.uk/media-library/sites/quadas/migrated/documents/background-doc.pdf.

[18] Borowsky AD, Glassy EF, Wallace WD (2020). Digital whole slide imaging compared with light microscopy for primary diagnosis in surgical pathology. Arch Pathol Lab Med.

[19] Jukić DM, Drogowski LM, Martina J, Parwani AV (2011). Clinical examination and validation of primary diagnosis in anatomic pathology using whole slide digital images. Arch Pathol Lab Med.

[20] Larghi A, Fornelli A, Lega S (2019). Concordance, intra- and inter-observer agreements between light microscopy and whole slide imaging for samples acquired by EUS in pancreatic solid lesions. Dig Liver Dis.

[21] Saco A, Diaz A, Hernandez M (2017). Validation of whole-slide imaging in the primary diagnosis of liver biopsies in a university hospital. Dig Liver Dis.

[22] van der Post RS, van der Laak JA, Sturm B, Clarijs R, Schaafsma HE, van Krieken JH, Nap M (2013). The evaluation of colon biopsies using virtual microscopy is reliable. Histopathology.

[23] Al-Janabi S, Huisman A, Vink A, Leguit RJ, Offerhaus GJ, ten Kate FJ, van Diest PJ (2012). Whole slide images for primary diagnostics of gastrointestinal tract pathology: a feasibility study. Hum Pathol.

[24] Al-Janabi S, Huisman A, Nikkels PG, ten Kate FJ, van Diest PJ (2013). Whole slide images for primary diagnostics of paediatric pathology specimens: a feasibility study. J Clin Pathol.

[25] Arnold MA, Chenever E, Baker PB (2015). The College of American Pathologists guidelines for whole slide imaging validation are feasible for pediatric pathology: a pediatric pathology practice experience. Pediatr Dev Pathol.

[26] Loughrey MB, Kelly PJ, Houghton OP (2015). Digital slide viewing for primary reporting in gastrointestinal pathology: a validation study. Virchows Arch.

[27] Mills AM, Gradecki SE, Horton BJ (2018). Diagnostic efficiency in digital pathology: a comparison of optical versus digital assessment in 510 surgical pathology cases. Am J Surg Pathol.

[28] Mukhopadhyay S, Feldman MD, Abels E (2018). Whole slide imaging versus microscopy for primary diagnosis in surgical pathology: a multicenter blinded randomized noninferiority study of 1992 cases (pivotal study). Am J Surg Pathol.

[29] Rao V, Kumar R, Rajaganesan S (2021). Remote reporting from home for primary diagnosis in surgical pathology: a tertiary oncology center experience during the COVID-19 pandemic. J Pathol Inform.

[30] Samuelson MI, Chen SJ, Boukhar SA (2021). Rapid validation of whole-slide imaging for primary histopathology diagnosis. Am J Clin Pathol.

[31] Snead DR, Tsang YW, Meskiri A (2016). Validation of digital pathology imaging for primary histopathological diagnosis. Histopathology.

[32] Tabata K, Mori I, Sasaki T (2017). Whole-slide imaging at primary pathological diagnosis: validation of whole-slide imaging-based primary pathological diagnosis at twelve Japanese academic institutes. Pathol Int.

[33] Thrall MJ, Wimmer JL, Schwartz MR (2015). Validation of multiple whole slide imaging scanners based on the guideline from the College of American Pathologists Pathology and Laboratory Quality Center. Arch Pathol Lab Med.

[34] Villa I, Mathieu MC, Bosq J (2018). Daily biopsy diagnosis in surgical pathology: concordance between light microscopy and whole-slide imaging in real-life conditions. Am J Clin Pathol.

[35] Holger Lange (2011). Digital pathology: a regulatory overview. Lab Med.

[36] Bauer TW, Schoenfield L, Slaw RJ, Yerian L, Sun Z, Henricks WH (2013). Validation of whole slide imaging for primary diagnosis in surgical pathology. Arch Pathol Lab Med.

[37] Campbell WS, Lele SM, West WW, Lazenby AJ, Smith LM, Hinrichs SH (2012). Concordance between whole-slide imaging and light microscopy for routine surgical pathology. Hum Pathol.

[38] Chantziantoniou N, Mukherjee M, Donnelly AD, Pantanowitz L, Austin RM (2018). Digital applications in cytopathology: problems, rationalizations, and alternative approaches. Acta Cytol.

[39] Hanna MG, Reuter VE, Hameed MR (2019). Whole slide imaging equivalency and efficiency study: experience at a large academic center. Mod Pathol.

